# Teacher emotional support and student well-being in higher education: a sequential motivational process via need satisfaction and autonomous motivation

**DOI:** 10.3389/fpsyg.2026.1851103

**Published:** 2026-07-08

**Authors:** Wei Liu, Shuangwen Zhao

**Affiliations:** 1 José Rizal University, Mandaluyong City, Philippines; 2 Rizal Technological University, Mandaluyong City, Philippines

**Keywords:** autonomous motivation, basic psychological need satisfaction, higher education, psychological well-being, self-determination theory, teacher emotional support

## Abstract

**Objective:**

This study examined whether teacher emotional support was associated with university students’ psychological well-being through basic psychological need satisfaction, autonomous motivation, and controlled motivation in Chinese higher education.

**Methods:**

Cross-sectional survey data from 445 Chinese undergraduate students were analyzed using latent-variable structural equation modeling. Gender and academic year were included as covariates, and indirect effects were evaluated using 5,000 bootstrap resamples.

**Results:**

The structural model demonstrated excellent fit, χ^2^(313) = 357.39, CFI = 0.992, TLI = 0.991, RMSEA = 0.018, and SRMR = 0.033, and explained 32.5% of the variance in psychological well-being. The sequential indirect association through basic psychological need satisfaction and autonomous motivation was significant (standardized effect = 0.155, bias-corrected 95% CI [0.092, 0.229]). In contrast, the indirect association through need satisfaction alone and the corresponding pathway through controlled motivation were not significant. Controlled motivation was not significantly associated with psychological well-being (β = 0.018, *p* = 0.720), and the direct association between teacher emotional support and psychological well-being was also non-significant (β = 0.087, *p* = 0.246).

**Conclusion:**

Teacher emotional support was associated with psychological well-being through a significant sequential motivational pathway involving basic psychological need satisfaction and autonomous motivation, supporting the relevance of motivational internalization processes in Chinese higher education.

## Introduction

University students’ psychological well-being has become an increasingly important concern in higher education systems worldwide, particularly as academic environments grow more competitive and demanding. Many university students experience elevated levels of academic stress, emotional exhaustion, and psychological strain during their studies (Pascoe et al., 2020; Widlund et al., 2021; Ning et al., 2024). These challenges may undermine students’ academic engagement, motivational functioning, and broader psychological adjustment, potentially affecting both academic persistence and long-term well-being (Asikainen et al., 2022). In highly competitive higher education systems such as China, intensified academic competition, graduate employment pressure, and the widely discussed phenomenon of “involution” (内卷)—a context in which individuals invest progressively greater effort in increasingly intense competition without achieving proportionate gains—are associated with heightened psychological strain and emotional fatigue among university students (Ning et al., 2024; Yan et al., 2022; Zhang et al., 2025). As Chinese university students increasingly navigate environments characterized by strong performance expectations and continuous social comparison, promoting psychologically sustainable forms of academic functioning has become an important concern for both educators and policymakers.

Teacher–student relationships, and particularly teacher emotional support, represent an important contextual factor shaping students’ psychological experiences in educational settings. Teacher support broadly refers to the extent to which teachers provide encouragement, guidance, and interpersonal care that are associated with students’ learning and development (Reeve and Cheon, 2021). Within this broader construct, teacher emotional support reflects the relational dimension of teacher–student interaction and includes teachers’ responsiveness, encouragement, interpersonal sensitivity, and emotional availability. Such interactions may foster psychologically supportive learning conditions for students. Previous research has shown that supportive teacher–student relationships are positively associated with students’ engagement, motivation, and academic achievement (Roorda et al., 2017; Jang et al., 2022; Huang et al., 2024). Although much of this research has focused on school contexts, emerging evidence suggests that teacher–student relationships remain highly relevant in higher education, where students must regulate their learning more independently (Jeno et al., 2023; Xu et al., 2023). Although teacher support is multidimensional and may also include instrumental or academic support related to learning strategies and cognitive guidance, the present study focuses specifically on emotional support because psychological well-being represents a primarily affective and motivational outcome. Emotional support characterized by empathy, warmth, and interpersonal understanding may therefore be particularly relevant to students’ psychological functioning in higher education settings. This focus does not imply that instrumental support is unimportant in higher education, but rather that emotional support may demonstrate more immediate relevance to students’ psychological functioning under sustained evaluative pressure.

Self-Determination Theory (SDT) provides a useful framework for understanding how supportive learning environments are associated with students’ motivation and psychological functioning (Ryan and Deci, 2020; Vansteenkiste et al., 2020). According to SDT, the satisfaction of three basic psychological needs: autonomy, competence, and relatedness—is positively associated with autonomous motivation and psychological well-being. Autonomous motivation reflects engagement that is personally endorsed or intrinsically valued, whereas controlled motivation reflects engagement driven by external pressures or obligations (Ryan and Deci, 2020). Previous studies suggest that autonomous motivation is strongly associated with adaptive outcomes and psychological well-being, whereas controlled motivation is often weakly related or unrelated to well-being (Howard et al., 2021; Bureau et al., 2022; Guay, 2022), as controlled forms of motivation may be shaped by externally regulating conditions beyond supportive teacher–student relationships. From this perspective, teacher emotional support may be associated with students’ well-being by supporting their psychological needs and shaping the quality of their motivation.

Within the SDT framework, basic psychological need satisfaction represents an important condition associated with motivational regulation and adaptive functioning. When students experience autonomy, competence, and relatedness, they are more likely to internalize academic goals and engage in learning activities with a stronger sense of personal endorsement (Howard et al., 2021; Vergara-Morales and Del Valle, 2021; Yang et al., 2024). This pattern is consistent with the Self-Concordance Model, which proposes that goals aligned with individuals’ values are associated with sustained effort and psychological well-being (Sheldon and Elliot, 1999). According to this perspective, supportive educational environments are associated with psychological need satisfaction, which in turn relates to more autonomous forms of motivational regulation and psychological well-being. This theoretically ordered pattern provides the conceptual basis for examining the proposed serial mediation model in the present study.

Despite growing evidence on teacher support, psychological need satisfaction, and student motivation, important gaps remain in understanding how these constructs jointly explain psychological well-being in higher education. First, although teacher support, psychological need satisfaction, and motivational processes have been widely studied, relatively few studies have examined how these constructs operate together within an integrated model explaining university students’ psychological well-being. Existing research has often focused on direct associations, providing limited insight into the theoretically ordered relationships among supportive teaching, motivational regulation, and psychological well-being. Second, although SDT has been widely applied in school contexts, fewer studies have examined how these relationships operate in higher education, where students face more complex academic demands and must regulate learning more independently. Third, relatively limited attention has been given to these relationships within highly competitive non-Western higher education systems such as China. In educational environments characterized by strong performance expectations and academic competition, examining the role of emotional support may provide additional insight into how supportive relational conditions are associated with students’ motivational functioning and psychological well-being.

Addressing these gaps, the present study draws on Self-Determination Theory to examine whether and how teacher emotional support is associated with psychological well-being through psychological need satisfaction and autonomous motivation within highly competitive Chinese higher education. Rather than treating emotional support as universally protective, the study proposes that its psychological relevance may depend on whether supportive interactions facilitate need satisfaction and motivational internalization. This sequential ordering is consistent with SDT assumptions that supportive contextual conditions are first internalized through psychological need satisfaction before shaping motivational regulation and psychological functioning (Ryan and Deci, 2020). Empirical support for this ordering has been provided in higher education contexts: Jeno et al. (2023) showed that autonomy-supportive teaching was associated with need satisfaction and autonomous motivation, Howard et al. (2021) meta-analytically confirmed that need satisfaction served as a proximal antecedent of autonomous motivation and showed stronger associations with well-being than controlled regulation, and Vergara-Morales and Del Valle (2021) demonstrated that supportive conditions were indirectly associated with psychological outcomes through need satisfaction and self-determined motivation. By examining these theoretically ordered relationships within Chinese higher education, the study extends SDT research in a highly competitive educational context and provides insight into the conditions under which emotionally supportive teaching remains psychologically relevant.

## Theoretical framework

### The direct relationship between teacher emotional support and psychological well-being

Within Self-Determination Theory (SDT), teacher emotional support represents a contextual resource that may be positively associated with students’ psychological well-being. Emotionally supportive interactions characterized by warmth, empathy, respect, and responsiveness may strengthen students’ sense of psychological security and interpersonal belonging, thereby contributing to more adaptive psychological functioning (Ryan and Deci, 2020; Federici and Skaalvik, 2014). In higher education, where students are expected to regulate their learning under substantial academic and evaluative demands, supportive teacher–student relationships may remain relevant to students’ motivation and well-being (Jeno et al., 2023). SDT further suggests that the association between supportive contextual conditions and psychological functioning may operate not only directly but also through psychological need satisfaction and the quality of motivational regulation (Howard et al., 2021; Bureau et al., 2022). From this perspective, the psychological relevance of teacher emotional support may be partly explained by whether supportive relational experiences are associated with greater autonomy, competence, relatedness, and more self-endorsed forms of academic engagement. Although teacher support may also include instrumental or academic dimensions, the present study focuses specifically on emotional support because it is theoretically more proximal to students’ affective and relational experiences.

### Psychological need satisfaction as the first mediating mechanism

Beyond its broader association with psychological well-being, teacher emotional support may be associated with psychological well-being partly through basic psychological need satisfaction. According to SDT, the satisfaction of three core psychological needs—autonomy, competence, and relatedness—provides the psychological foundation for adaptive functioning and well-being (Ryan and Deci, 2020; Vansteenkiste et al., 2020). Emotionally supportive teacher–student interactions may strengthen students’ perceived autonomy by reducing pressure-based regulation, affirm students’ sense of competence through encouragement and responsiveness, and satisfy relatedness needs through psychologically meaningful interpersonal interactions (Jeno et al., 2023). When students experience these needs as fulfilled, they are more likely to report greater psychological well-being. Previous research consistently demonstrates that psychological need satisfaction is positively associated with adaptive psychological outcomes in educational settings (Howard et al., 2021; Vergara-Morales and Del Valle, 2021). Accordingly, basic psychological need satisfaction is proposed as the first mediating mechanism linking teacher emotional support to psychological well-being.

### Motivational regulation as the second mediating mechanism

The pathway from teacher emotional support to psychological well-being may extend further through the quality of students’ motivational regulation. Within SDT and the Self-Concordance Model (Sheldon and Elliot, 1999), psychological need satisfaction is associated with greater internalization of academic goals and more autonomous forms of motivational regulation. Autonomous motivation reflects engagement driven by personal interest and self-endorsed values, whereas controlled motivation reflects engagement driven by external demands, obligations, or pressure-based regulation (Ryan and Deci, 2020). When students experience their basic psychological needs as satisfied, they are more likely to engage in academic activities in a self-determined manner, which in turn is associated with greater psychological well-being. Previous studies have consistently shown that autonomous motivation is positively associated with adaptive educational outcomes and psychological well-being, whereas controlled motivation tends to show weaker or non-significant associations with well-being (Howard et al., 2021; Bureau et al., 2022; Guay, 2022). In highly competitive educational contexts, controlled motivation may be less dependent on supportive teacher–student relationships, which may explain its comparatively weaker expected association with psychological well-being. Accordingly, autonomous motivation is proposed as the second mediating mechanism in the sequential pathway from teacher emotional support through psychological need satisfaction to psychological well-being, whereas controlled motivation is expected to demonstrate a comparatively weaker association with well-being.

### Research model and hypotheses

**Figure 1:**
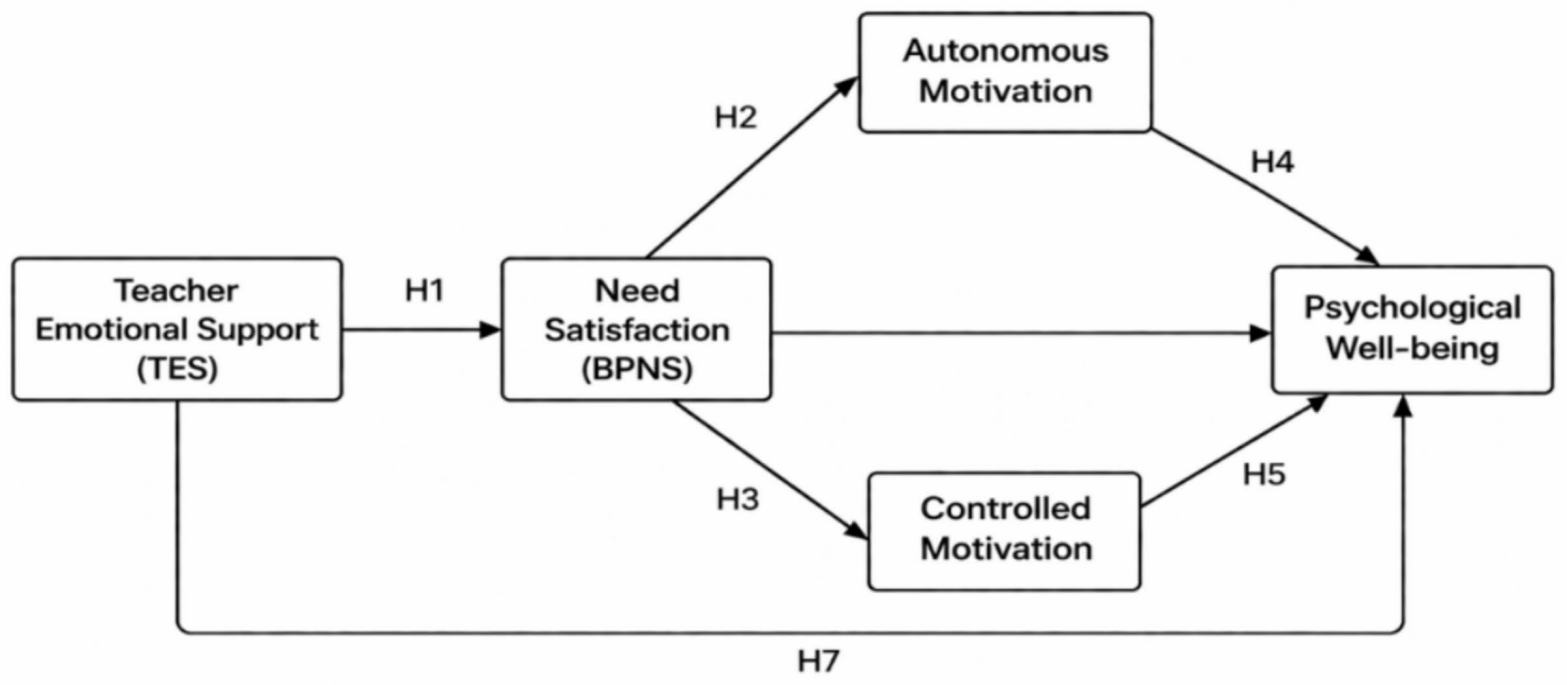
Conceptual research model of the relationships among teacher emotional support, basic psychological need satisfaction, autonomous motivation, controlled motivation, and psychological well-being.

Building on the theoretical framework, this study proposes a serial mediation model in which teacher emotional support is associated with psychological well-being through basic psychological need satisfaction and autonomous motivation, while also examining the role of controlled motivation. The model conceptualizes psychological well-being as associated with theoretically ordered motivational relationships rather than independent predictors examined in isolation (Figure 1). Specifically, teacher emotional support is expected to be positively associated with need satisfaction, which in turn relates to autonomous motivation and psychological well-being. In contrast, controlled motivation is expected to demonstrate weaker or non-significant associations with psychological well-being.

*H1*: Teacher emotional support is positively associated with basic psychological need satisfaction.

*H2*: Basic psychological need satisfaction is positively associated with autonomous motivation.

*H3*: Basic psychological need satisfaction is associated with controlled motivation.

*H4*: Autonomous motivation is positively associated with psychological well-being.

*H5*: The association between autonomous motivation and psychological well-being is stronger than the corresponding association between controlled motivation and psychological well-being.

*H6*: Teacher emotional support is indirectly associated with psychological well-being through the sequential mediation of basic psychological need satisfaction and autonomous motivation.

*H7*: Teacher emotional support is positively associated with psychological well-being after accounting for the proposed indirect pathways.

## Method

### Research design

This study employed a cross-sectional quantitative research design using structural equation modeling (SEM) to examine the relationships among teacher emotional support, basic psychological need satisfaction, autonomous motivation, controlled motivation, and psychological well-being among university students. Structural equation modeling was selected because it is widely used for examining theoretically specified relationships among latent psychological constructs (Hair et al., 2019; Kline, 2016). The study was grounded in Self-Determination Theory (SDT) and the Self-Concordance Model, which conceptualize psychological well-being as associated with supportive relational conditions, psychological need satisfaction, and motivational regulation.

A cross-sectional design was considered appropriate because the study aimed to examine theoretically specified associations among psychological constructs within a higher education context rather than establish temporal or causal relationships. Consistent with recommendations for cross-sectional mediation research, the findings are interpreted as correlational associations rather than causal effects (Maxwell and Cole, 2007). The final structural model also included gender and academic year as covariates. Specific indirect associations were estimated for the pathways through basic psychological need satisfaction alone, through basic psychological need satisfaction and autonomous motivation, and through basic psychological need satisfaction and controlled motivation.

### Participants and sampling procedure

The participants consisted of 445 undergraduate students enrolled in Chinese universities. Students were recruited from several higher education institutions through convenience-based institutional access combined with voluntary participant recruitment. Eligibility criteria required participants to be currently enrolled undergraduate students and actively attending university courses during the data collection period.

The sample included students from different academic disciplines and year levels to provide broader representation of university learning experiences within Chinese higher education. The final sample size of 445 was considered adequate for SEM analysis and exceeded commonly recommended minimum thresholds for latent variable modeling (Hair et al., 2019; Jackson, 2003; Kline, 2016). The final dataset contained complete responses from 445 participants. No missing values were identified across the 25 measurement items or the demographic variables included in the structural model. Accordingly, all 445 cases were retained, and no deletion or data-imputation procedure was required.

Data collection commenced on 29 December 2025 and was completed in February 2026. An anonymous online questionnaire was distributed through university communication platforms and student networks. Participation was voluntary, and respondents were informed that their responses would remain confidential and would be used exclusively for academic research purposes.

### Measures

Because the original instruments were developed in English, all questionnaire items were translated into Chinese using a back-translation procedure following Brislin (1970). Two bilingual researchers independently translated and back-translated the items to ensure semantic equivalence across language versions.

### Teacher emotional support

Teacher emotional support was measured using items adapted from previous studies on emotional teacher support and supportive teacher–student relationships (Federici and Skaalvik, 2014). The construct assessed students’ perceptions of teachers’ empathy, encouragement, warmth, respect, and emotional understanding. Sample items included statements reflecting supportive interpersonal interactions and teachers’ responsiveness to students’ emotional and academic concerns.

Responses were measured using a five-point Likert scale ranging from 1 (“strongly disagree”) to 5 (“strongly agree”), with higher scores indicating stronger perceived teacher emotional support.

### Basic psychological need satisfaction

Basic psychological need satisfaction was measured based on the three core psychological needs identified within Self-Determination Theory: autonomy, competence, and relatedness (Ryan and Deci, 2017). The instrument was adapted from the Basic Psychological Need Satisfaction and Frustration Scale (BPNSFS; Chen et al., 2015) and assessed students’ overall perceptions of psychological fulfillment within their university learning experiences.

Autonomy reflected students’ perceptions of volition and self-direction in learning activities. Competence reflected students’ feelings of effectiveness and capability in academic tasks. Relatedness reflected students’ feelings of connection and belonging within the educational environment.

Responses were measured using a five-point Likert scale ranging from 1 (“strongly disagree”) to 5 (“strongly agree”), with higher scores indicating greater psychological need satisfaction.

### Autonomous motivation and controlled motivation

Autonomous motivation and controlled motivation were measured using adapted items based on motivational regulation frameworks developed within Self-Determination Theory research (Ryan and Connell, 1989; Howard et al., 2021). Autonomous motivation assessed engagement driven by personal interest, personal importance, and self-endorsed values. Controlled motivation assessed engagement driven by external demands, pressure, obligation, or avoidance of negative consequences.

Consistent with SDT, autonomous and controlled motivation were conceptualized as distinct forms of motivational regulation rather than opposite ends of a single continuum.

Responses were measured using a five-point Likert scale ranging from 1 (“strongly disagree”) to 5 (“strongly agree”). Higher scores indicated stronger endorsement of each motivational orientation.

### Psychological well-being

Psychological well-being was assessed using items conceptually aligned with eudaimonic approaches to well-being emphasizing positive functioning, meaning, and psychological fulfillment (Ryff, 1989; Sheldon and Prentice, 2019). The construct reflected students’ positive psychological functioning, emotional adjustment, and overall psychological wellness within the university context.

Responses were measured using a five-point Likert scale ranging from 1 (“strongly disagree”) to 5 (“strongly agree”), with higher scores indicating higher levels of psychological well-being.

### Instrument evaluation

Before the main analysis, the adapted questionnaire was reviewed for linguistic clarity, conceptual relevance, and consistency with the definitions of the five focal constructs. The psychometric properties of the measures were subsequently evaluated using the final analytical sample.

Internal consistency was assessed using Cronbach’s alpha and composite reliability. Convergent validity was evaluated using standardized factor loadings and average variance extracted. Discriminant validity was assessed using the heterotrait–monotrait ratio of correlations (Henseler et al., 2015). A five-factor confirmatory factor analysis was estimated using all 25 observed indicators, with five indicators specified for each latent construct. Alternative measurement models were also estimated by combining theoretically related constructs and comparing their fit with that of the hypothesized five-factor model. The corresponding reliability and validity results are reported in the Results section.

### Common method bias assessment

Because all focal constructs were assessed using self-report measures within the same survey administration, both procedural and statistical approaches were used to address potential common method bias. Procedural measures included anonymous participation, confidentiality assurances, and the separation of construct sections within the questionnaire to reduce evaluation apprehension and response-consistency motives (Podsakoff et al., 2003).

Statistically, Harman’s single-factor test was conducted by entering all 25 measurement items into an unrotated principal component analysis and examining the proportion of variance accounted for by the first unrotated component. In addition, a single-factor confirmatory factor model was estimated and compared with the hypothesized five-factor measurement model. The results of these analyses are reported in the Results section.

### Data analysis procedure

Data screening and descriptive analyses were conducted using SPSS 26.0. The dataset was examined for missing values, and the distributions of the five composite variables were evaluated using means, standard deviations, skewness, and kurtosis. Pearson correlations were calculated to examine the zero-order associations among the focal constructs.

The measurement and structural models were estimated using maximum likelihood estimation. Maximum likelihood estimation was retained because the observed skewness and kurtosis values did not indicate severe univariate non-normality. Nevertheless, because indirect effects commonly have non-normal sampling distributions, nonparametric bootstrap procedures were used for statistical inference regarding the indirect effects.

The measurement model specified five correlated latent constructs and 25 observed indicators, with five indicators assigned to each construct. Internal consistency was evaluated using Cronbach’s alpha and composite reliability. Convergent validity was assessed using standardized factor loadings and average variance extracted, while discriminant validity was examined using the heterotrait–monotrait ratio. The hypothesized five-factor model was additionally compared with alternative models in which theoretically related constructs were combined.

Model fit was evaluated using the chi-square statistic, the chi-square-to-degrees-of-freedom ratio, the comparative fit index, the Tucker–Lewis index, the root mean square error of approximation, and the standardized root mean square residual. CFI and TLI values of 0.90 or higher and RMSEA and SRMR values of 0.08 or lower were interpreted as indicating acceptable fit. Values closer to 0.95 for CFI and TLI and 0.06 for RMSEA were considered indicative of stronger fit (Hu and Bentler, 1999).

The structural model specified paths from teacher emotional support to basic psychological need satisfaction and psychological well-being; from basic psychological need satisfaction to autonomous motivation, controlled motivation, and psychological well-being; and from autonomous and controlled motivation to psychological well-being. Gender and academic year were included as covariates in the equations predicting basic psychological need satisfaction, autonomous motivation, controlled motivation, and psychological well-being. Gender was dummy-coded with female participants as the reference category. Academic year was coded from 1 to 4 and treated as an ordinal covariate.

Because H5 concerned whether the association between autonomous motivation and psychological well-being was stronger than the corresponding association involving controlled motivation, the two structural coefficients were compared using a likelihood-ratio test. The unconstrained model was compared with a nested model in which the two pathways were constrained to equality.

Specific indirect effects were evaluated for three pathways: teacher emotional support → basic psychological need satisfaction → psychological well-being; teacher emotional support → basic psychological need satisfaction → autonomous motivation → psychological well-being; and teacher emotional support → basic psychological need satisfaction → controlled motivation → psychological well-being. The total indirect effect was calculated as the sum of these three specific indirect effects. Statistical inference was based on 5,000 nonparametric bootstrap resamples and 95% bias-corrected confidence intervals. An indirect effect was considered statistically significant when its confidence interval did not include zero (Preacher and Hayes, 2008).

Because the study employed a cross-sectional design, all structural and indirect pathways were interpreted as theoretically specified associations rather than evidence of temporal or causal effects.

### Ethical considerations

This anonymous, minimal-risk questionnaire study was conducted in regular university educational settings and involved no intervention, clinical procedure, behavioral manipulation, or collection of personally identifiable information. Participation was voluntary, and electronic informed consent was obtained before questionnaire completion. Participants were informed of the study purpose, the confidentiality of their responses, and their right to withdraw at any time without penalty. Under the applicable institutional guidelines, formal ethics committee review was not required for anonymous, minimal-risk educational survey research.

## Results

### Data screening and common method bias

The final analytical sample consisted of 445 participants. Complete responses were available for all 25 measurement items, and no missing values were identified. All 445 cases were therefore retained for analysis, and no deletion or imputation procedure was required.

Because all focal constructs were assessed through self-report questionnaires administered within the same survey, Harman’s single-factor test was conducted as an initial assessment of potential common method bias. All 25 measurement items were entered into an unrotated principal component analysis. The first unrotated factor accounted for 32.47% of the total variance, below the commonly referenced 40% threshold. In addition, the single-factor confirmatory factor model demonstrated substantially poorer fit than the hypothesized five-factor measurement model. Taken together, these results indicated that a single common factor was unlikely to account for the observed covariance among the study variables.

### Descriptive statistics, distributional characteristics, and correlations

Table 1 presents the means, standard deviations, skewness, kurtosis, and zero-order correlations among the focal constructs. Teacher emotional support (*M* = 4.34, SD = 0.75), basic psychological need satisfaction (*M* = 4.29, SD = 0.85), autonomous motivation (*M* = 4.25, SD = 0.82), and psychological well-being (*M* = 4.31, SD = 0.78) were rated relatively highly on the five-point response scale. Controlled motivation had a comparatively lower mean score (*M* = 3.73, SD = 1.00).

**Table 1:** Descriptive statistics, distributional characteristics, and correlations.

Variable	*M*	SD	Skew	Kurt	1	2	3	4	5
1. Teacher emotional support	4.34	0.75	−1.44	1.82	–				
2. Basic psychological need satisfaction	4.29	0.85	−1.41	1.42	0.629***	–			
3. Autonomous motivation	4.25	0.82	−1.27	1.03	0.332***	0.525***	–		
4. Controlled motivation	3.73	1.00	−0.64	−0.41	0.196***	0.249***	0.140**	–	
5. Psychological well-being	4.31	0.78	−1.41	1.72	0.318***	0.423***	0.445***	0.132**	–

*N* = 445, **p < 0.01, ***p < 0.001.

The relatively high means and negative skewness values indicated some concentration of responses toward the upper end of the scale. However, the absolute skewness values ranged from 0.64 to 1.44, and the absolute kurtosis values ranged from 0.41 to 1.82. These values did not indicate severe univariate non-normality. Maximum likelihood estimation was therefore retained. To obtain more robust inference for the indirect effects without relying on the normality of their sampling distributions, statistical significance was additionally evaluated using 5,000 nonparametric bootstrap resamples.

Teacher emotional support was positively correlated with basic psychological need satisfaction (*r* = 0.629, *p* < 0.001), autonomous motivation (*r* = 0.332, *p* < 0.001), controlled motivation (*r* = 0.196, *p* < 0.001), and psychological well-being (*r* = 0.318, *p* < 0.001). Basic psychological need satisfaction was positively correlated with autonomous motivation (*r* = 0.525, *p* < 0.001), controlled motivation (*r* = 0.249, *p* < 0.001), and psychological well-being (*r* = 0.423, *p* < 0.001). Autonomous motivation was positively correlated with psychological well-being (*r* = 0.445, *p* < 0.001), whereas controlled motivation showed a comparatively weak positive correlation with psychological well-being (*r* = 0.132, *p* < 0.01).

### Measurement model

A five-factor confirmatory factor analysis was conducted before testing the structural relationships. Each latent construct—teacher emotional support, basic psychological need satisfaction, autonomous motivation, controlled motivation, and psychological well-being—was represented by five observed indicators, yielding a total of 25 measurement items.

As shown in Table 2, the hypothesized five-factor measurement model demonstrated excellent fit to the data, χ^2^(265) = 299.91, χ^2^/df = 1.13, CFI = 0.993, TLI = 0.993, RMSEA = 0.017, and SRMR = 0.033. All standardized factor loadings were statistically significant. The loading ranges were 0.672–0.773 for teacher emotional support, 0.801–0.836 for basic psychological need satisfaction, 0.713–0.763 for autonomous motivation, 0.679–0.730 for controlled motivation, and 0.723–0.771 for psychological well-being. These findings indicated that the 25 observed indicators adequately represented their respective latent constructs.

**Table 2:** Fit indices for the five-factor measurement model.

Fit index	Value
χ^2^	299.91
df	265
χ^2^/df	1.13
CFI	0.993
TLI	0.993
RMSEA	0.017
SRMR	0.033

### Reliability and convergent validity

Internal consistency reliability was evaluated using Cronbach’s alpha and composite reliability. As shown in Table 3, Cronbach’s alpha coefficients ranged from 0.833 to 0.910, while composite reliability values ranged from 0.833 to 0.911, indicating satisfactory reliability across the five constructs.

**Table 3:** Reliability and convergent validity.

Construct	Cronbach’s α	CR	AVE	Standardized loading range
Teacher emotional support	0.849	0.849	0.531	0.672–0.773
Basic psychological need satisfaction	0.910	0.911	0.672	0.801–0.836
Autonomous motivation	0.855	0.856	0.543	0.713–0.763
Controlled motivation	0.833	0.833	0.499	0.679–0.730
Psychological well-being	0.863	0.864	0.559	0.723–0.771

Average variance extracted values ranged from 0.499 to 0.672. Teacher emotional support, basic psychological need satisfaction, autonomous motivation, and psychological well-being exceeded the conventional 0.50 benchmark. Controlled motivation had an AVE of 0.499, which was marginally below 0.50. However, its composite reliability was 0.833, and all five standardized loadings were statistically significant and exceeded 0.67. The convergent validity of controlled motivation was therefore considered marginal but acceptable.

### Discriminant validity

Discriminant validity was assessed using the heterotrait–monotrait ratio. As shown in Table 4, the HTMT values ranged from 0.157 to 0.716, and all values were below the conservative 0.85 threshold. The highest value was observed between teacher emotional support and basic psychological need satisfaction (HTMT = 0.716), indicating that these theoretically related constructs remained empirically distinguishable.

**Table 4:** Heterotrait–Monotrait ratios.

Construct	TES	BPNS	AM	CM	PWB
TES	–				
BPNS	0.716	–			
AM	0.387	0.593	–		
CM	0.232	0.286	0.165	–	
PWB	0.372	0.476	0.519	0.157	–

TES, teacher emotional support; BPNS, basic psychological need satisfaction; AM, autonomous motivation; CM, controlled motivation; PWB, psychological well-being.

### Alternative measurement models

A series of alternative measurement models was estimated to further assess the empirical distinctiveness of the five constructs. As shown in Table 5, the hypothesized five-factor model provided substantially better fit than all competing models.

**Table 5:** Comparison of the hypothesized and alternative measurement models.

Model	χ^2^	df	χ^2^/df	CFI	TLI	RMSEA	SRMR
Five-factor model	299.91	265	1.13	0.993	0.993	0.017	0.033
TES + BPNS merged	625.29	269	2.32	0.933	0.926	0.055	0.050
BPNS + AM merged	818.48	269	3.04	0.897	0.885	0.068	0.068
TES + BPNS + AM merged	1172.66	272	4.31	0.831	0.814	0.086	0.081
Single-factor model	2480.39	275	9.02	0.587	0.550	0.134	0.131

Combining teacher emotional support and basic psychological need satisfaction reduced model fit to χ^2^(269) = 625.29, CFI = 0.933, TLI = 0.926, RMSEA = 0.055, and SRMR = 0.050. Combining basic psychological need satisfaction and autonomous motivation resulted in further deterioration, χ^2^(269) = 818.48, CFI = 0.897, TLI = 0.885, RMSEA = 0.068, and SRMR = 0.068. The model combining teacher emotional support, basic psychological need satisfaction, and autonomous motivation demonstrated poor fit, while the single-factor model showed clearly unacceptable fit. These comparisons provided additional support for the hypothesized five-factor measurement structure.

### Structural model and hypothesis testing

The hypothesized sequential latent-variable model was estimated using maximum likelihood estimation. Gender was dummy-coded with female as the reference category, and academic year was coded from 1 to 4 and treated as an ordinal covariate. Both variables were entered as controls in the equations predicting basic psychological need satisfaction, autonomous motivation, controlled motivation, and psychological well-being.

The structural model demonstrated excellent fit to the data, χ^2^(313) = 357.39, χ^2^/df = 1.14, CFI = 0.992, TLI = 0.991, RMSEA = 0.018, and SRMR = 0.033. The structural path estimates and hypothesis-testing results are summarized in Table 6.

**Table 6:** Structural model results.

Structural path	*B*	SE	β	*p*	95% CI for B	Path significance
TES → BPNS	0.705	0.056	0.709	<0.001	[0.596, 0.814]	Significant
BPNS → AM	0.549	0.052	0.587	<0.001	[0.447, 0.651]	Significant
BPNS → CM	0.331	0.063	0.291	<0.001	[0.208, 0.455]	Significant
BPNS → PWB	0.188	0.084	0.195	0.024	[0.024, 0.352]	Significant
AM → PWB	0.383	0.068	0.372	<0.001	[0.248, 0.517]	Significant
CM → PWB	0.015	0.043	0.018	0.720	[−0.069, 0.100]	Not significant
TES → PWB	0.083	0.072	0.087	0.246	[−0.057, 0.224]	Not significant

*N* = 445. B = unstandardized coefficient; SE = standard error; β = standardized coefficient. The confidence intervals refer to the unstandardized coefficients and were calculated from the maximum-likelihood estimates and their standard errors. Gender and academic year were included as control variables. Hypothesis decisions are reported in the accompanying text because H5 concerns a comparison between two structural paths rather than the statistical significance of the CM → PWB path alone.

Teacher emotional support was positively associated with basic psychological need satisfaction (*B* = 0.705, SE = 0.056, β = 0.709, *p* < 0.001), supporting H1. Basic psychological need satisfaction was positively associated with autonomous motivation (*B* = 0.549, SE = 0.052, β = 0.587, *p* < 0.001) and controlled motivation (*B* = 0.331, SE = 0.063, β = 0.291, *p* < 0.001), supporting H2 and H3.

Autonomous motivation was positively associated with psychological well-being (*B* = 0.383, SE = 0.068, β = 0.372, *p* < 0.001), supporting H4. Controlled motivation was not significantly associated with psychological well-being (*B* = 0.015, SE = 0.043, β = 0.018, *p* = 0.720). A likelihood-ratio test showed that the autonomous motivation–well-being path was significantly stronger than the controlled motivation–well-being path, Δχ^2^(1) = 22.09, *p* < 0.001. Therefore, H5 was supported.

Basic psychological need satisfaction retained a significant positive association with psychological well-being (*B* = 0.188, SE = 0.084, β = 0.195, *p* = 0.024). In contrast, the direct association between teacher emotional support and psychological well-being was not statistically significant after the mediators and control variables were included (*B* = 0.083, SE = 0.072, β = 0.087, *p* = 0.246). Consequently, H7 was not supported.

None of the paths from gender or academic year to the four endogenous constructs reached statistical significance. The model explained 50.6% of the variance in basic psychological need satisfaction, 35.1% of the variance in autonomous motivation, 8.6% of the variance in controlled motivation, and 32.5% of the variance in psychological well-being.

**Figure 2:**
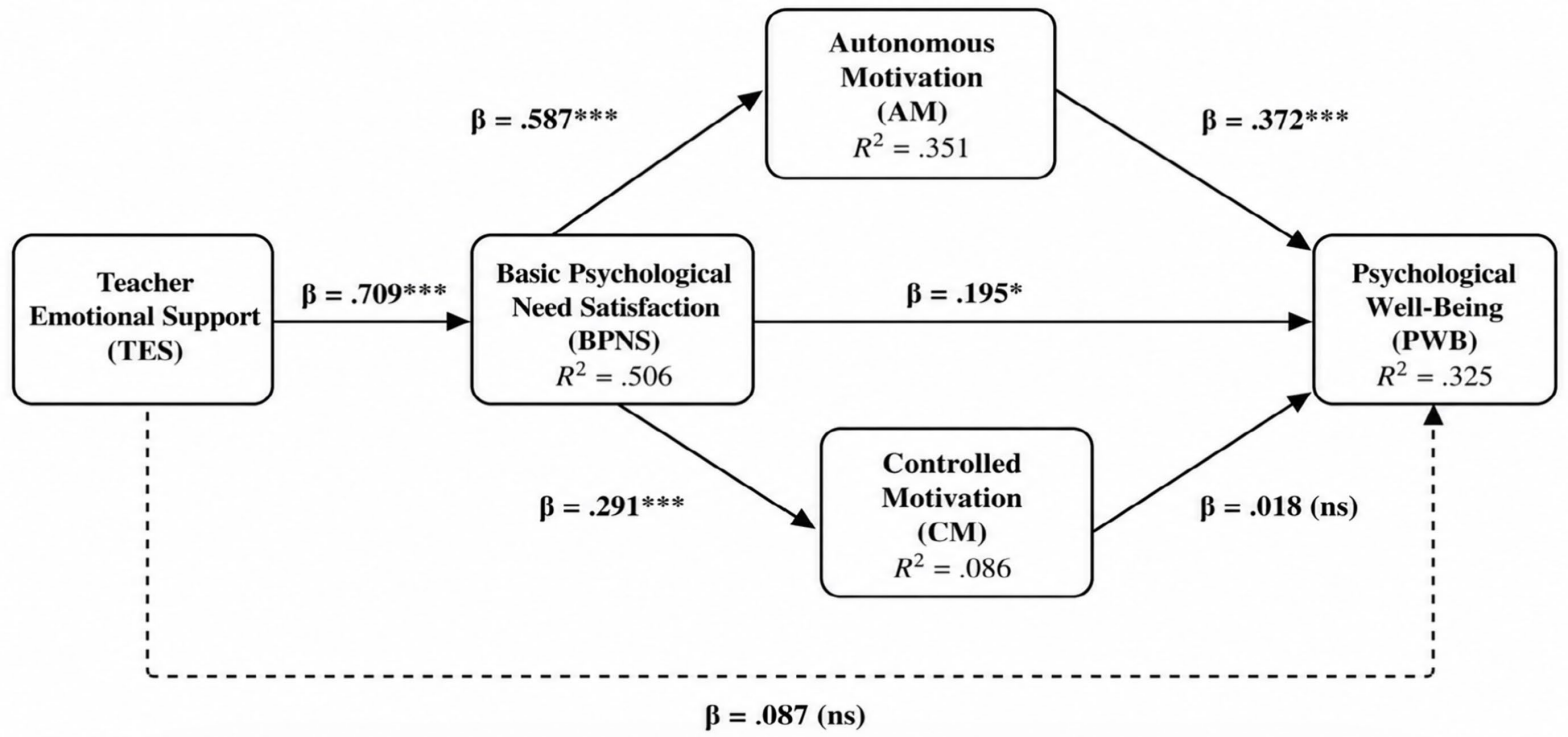
Final structural model. Values shown are standardized path coefficients (β). Solid lines represent the hypothesized structural paths, whereas the dashed line represents the direct association between teacher emotional support and psychological well-being. Gender and academic year were included as control variables but are omitted from the figure for clarity. *R*^2^ values indicate the proportion of variance explained in each endogenous construct. *p < 0.05. ***p < 0.001. ns, not significant.

Figure 2 presents the standardized path coefficients and the explained variance in the endogenous constructs.

### Bootstrap tests of the indirect effects

The specific indirect effects were evaluated using 5,000 nonparametric bootstrap resamples. All 5,000 bootstrap samples converged successfully, with no convergence failures. The bootstrapped specific and total indirect effects are presented in Table 7.

**Table 7:** Bootstrapped specific indirect effects of teacher emotional support on psychological well-being.

Indirect pathway	Unstandardized effect	Standardized effect	Boot SE	Percentile 95% CI	Bias-corrected 95% CI	Result
TES → BPNS → PWB	0.133	0.138	0.073	[−0.011, 0.279]	[−0.011, 0.278]	Not significant
TES → BPNS → AM → PWB	0.148	0.155	0.033	[0.088, 0.219]	[0.092, 0.229]	Significant
TES → BPNS → CM → PWB	0.004	0.004	0.010	[−0.016, 0.026]	[−0.015, 0.027]	Not significant
Total indirect effect	0.285	0.297	0.066	[0.160, 0.423]	[0.166, 0.428]	Significant

*N* = 445. Indirect effects were evaluated using 5,000 nonparametric bootstrap resamples, with no convergence failures. Bootstrap standard errors and confidence intervals refer to the unstandardized indirect effects; standardized estimates are reported separately as effect-size indices. An indirect effect was considered statistically significant when its 95% bias-corrected confidence interval excluded zero. Gender and academic year were included as control variables.

The specific indirect association between teacher emotional support and psychological well-being through basic psychological need satisfaction alone was not statistically significant (*B* = 0.133, standardized effect = 0.138, Boot SE = 0.073, BC 95% CI [−0.011, 0.278]). Although the BPNS → PWB structural path was significant, the confidence interval for the corresponding indirect product included zero after autonomous and controlled motivation were included as downstream mediators.

The sequential indirect association through basic psychological need satisfaction and autonomous motivation was statistically significant (*B* = 0.148, standardized effect = 0.155, Boot SE = 0.033, BC 95% CI [0.092, 0.229]). This finding supported H6 and indicated that teacher emotional support was indirectly associated with psychological well-being through the sequential pathway involving basic psychological need satisfaction and autonomous motivation.

The corresponding sequential indirect association through controlled motivation was not statistically significant (*B* = 0.004, standardized effect = 0.004, Boot SE = 0.010, BC 95% CI [−0.015, 0.027]). The total indirect association was statistically significant (B = 0.285, standardized effect = 0.297, Boot SE = 0.066, BC 95% CI [0.166, 0.428]).

Overall, the statistically supported indirect pathway operated through basic psychological need satisfaction and autonomous motivation, whereas the residual direct association was not statistically significant.

## Discussion

### Integration of the main findings

The present study examined whether teacher emotional support was associated with university students’ psychological well-being through basic psychological need satisfaction, autonomous motivation, and controlled motivation. The results showed that the sequential indirect association through basic psychological need satisfaction and autonomous motivation was statistically significant. In contrast, the specific indirect association through basic psychological need satisfaction alone, the parallel sequential association through controlled motivation, and the residual direct association between teacher emotional support and psychological well-being were not statistically significant.

These findings are consistent with an SDT-based account in which emotionally supportive teaching is associated with students’ psychological need fulfillment and more self-endorsed academic motivation, which are in turn associated with psychological well-being (Ryan and Deci, 2020; Sheldon and Elliot, 1999). Supportive interactions characterized by empathy, respect, encouragement, and responsiveness may correspond with stronger experiences of autonomy, competence, and relatedness, while autonomous motivation may reflect the extent to which academic engagement is personally valued and endorsed. Because the study was cross-sectional, this ordering should be interpreted as a theoretically specified pattern of associations rather than evidence of temporal or causal mediation.

Basic psychological need satisfaction was positively associated with both autonomous motivation and psychological well-being. However, the confidence interval for the TES → BPNS → PWB indirect effect included zero. This is not inconsistent with the significant BPNS → PWB path because the significance of an individual component path does not guarantee the significance of the complete indirect product. The supported sequential pathway through need satisfaction and autonomous motivation should therefore be interpreted as a statistically supported indirect association within the specified model rather than evidence that autonomous motivation is a necessary mechanism or that this pathway was significantly stronger than the BPNS-only pathway.

Teacher emotional support and basic psychological need satisfaction were moderately strongly associated but remained conceptually and empirically distinct. Teacher emotional support represents a relational feature of the educational environment, whereas need satisfaction reflects students’ internal experiences of autonomy, competence, and relatedness. The HTMT results and alternative measurement-model comparisons supported this distinction. Thus, the findings are consistent with the proposition that emotionally supportive teacher–student relationships may represent one relational condition associated with students’ experiences of need fulfillment without implying that the two constructs are interchangeable.

### Autonomous and controlled motivation

A central contribution of the study lies in differentiating the autonomous and controlled motivational pathways within the association between teacher emotional support and psychological well-being. Autonomous motivation was positively associated with psychological well-being and formed part of the statistically significant sequential indirect pathway. Controlled motivation, by contrast, was not uniquely associated with psychological well-being after the other variables were included. The autonomous motivation–well-being path was also significantly stronger than the corresponding controlled motivation–well-being path.

This pattern is consistent with the SDT distinction between engagement based on personal endorsement and engagement driven by external demands or internal pressure (Ryan and Deci, 2020; Howard et al., 2021; Guay, 2022). Controlled motivation may remain relevant to other academic outcomes, but it was not independently associated with psychological well-being in the present model. Autonomous and controlled motivation should not be treated as mutually exclusive. Students may value their studies while simultaneously experiencing pressure related to grades, family expectations, institutional requirements, or employment competition.

The model explained only 8.6% of the variance in controlled motivation, suggesting that important antecedents were not represented. Future research should examine broader influences such as institutional performance pressure, interpersonal expectations, and employment-related concerns. These factors may be especially relevant in Chinese higher education, where prior research has described increasing academic competition, graduate employment uncertainty, and “involution” (Ning et al., 2024; Yan et al., 2022). However, these contextual conditions were not directly measured and should therefore be treated as interpretive background rather than as tested explanations of the present findings.

### Theoretical and practical implications

The study contributes to SDT research by showing that the relational relevance of teacher emotional support should be interpreted in conjunction with the quality of students’ motivational regulation. The findings suggest that supportive teacher–student relationships may be associated with psychological well-being differently depending on whether students’ academic engagement is experienced as self-endorsed or pressure-based.

The study also extends research that has often focused on general teacher support or autonomy-supportive instruction by examining teacher emotional support as a distinct relational construct. The findings suggest that warmth, empathy, respect, encouragement, and responsiveness may be relevant to students’ psychological functioning through their associations with need fulfillment and autonomous motivation. They also provide additional evidence that SDT-consistent associations can be observed in a Chinese higher education sample. Nevertheless, comparative and longitudinal research is required before the stability of these relationships across cultural and institutional contexts can be established.

The most direct practical implications concern the quality of teacher–student interactions. Faculty members may consider using respectful communication, constructive encouragement, interpersonal warmth, and responsiveness to students’ academic and emotional concerns. Faculty-development programs may also distinguish these emotionally supportive practices from broader autonomy-supportive strategies, such as acknowledging students’ perspectives, explaining the rationale for academic requirements, and minimizing unnecessarily controlling language (Reeve and Cheon, 2021; Jang et al., 2022). These recommendations should remain cautious because the present design does not demonstrate that changing teacher behavior will cause improvements in motivation or psychological well-being.

### Limitations and future directions

Several limitations should be acknowledged. First, the cross-sectional design does not establish temporal ordering or causality, and alternative directional relationships remain possible (Maxwell and Cole, 2007). Longitudinal, multi-wave, and intervention-based studies are needed to test the proposed sequence over time. Second, all focal constructs were assessed using self-report measures administered within the same survey. Although procedural remedies, Harman’s single-factor test, and comparison with a single-factor CFA model were used, shared-method variance cannot be ruled out. Future research should incorporate teacher reports, observations, behavioral indicators, or temporally separated measurements. Third, several constructs showed relatively high means and negative skewness, indicating some concentration of responses toward the upper end of the scale. This potential ceiling-related compression may have reduced differentiation among respondents with relatively positive experiences. Wider response scales, alternative item wording, and more heterogeneous samples may improve measurement sensitivity. Fourth, basic psychological need satisfaction was modeled as a global construct, and teacher emotional support and need satisfaction were moderately strongly associated. Although the CFA, HTMT analysis, and alternative-model comparisons supported their empirical distinctiveness, Although the measurement analyses supported empirical distinctiveness, the same-source, cross-sectional design limited the strength of conclusions regarding the conceptual separation of contextual support and internal psychological experience. The study also did not permit separate examination of autonomy, competence, and relatedness. Future research should employ comprehensive need-specific measures, multi-source data, and longitudinal designs to clarify the distinct roles of the three needs and the conceptual boundary between relational support and need fulfillment.

Finally, the convenience sample was limited to Chinese undergraduate students, restricting population representativeness and broader generalizability. In addition, the study did not directly measure the institutional and sociocultural pressures used as contextual background, and the model explained relatively little variance in controlled motivation. Future studies should include more diverse student populations and directly assess institutional pressure, interpersonal expectations, and employment-related concerns.

## Conclusion

This study found that teacher emotional support was associated with Chinese university students’ psychological well-being through a statistically significant sequential indirect pathway involving basic psychological need satisfaction and autonomous motivation. In contrast, the corresponding pathway through controlled motivation and the residual direct association between teacher emotional support and psychological well-being were not statistically significant. By distinguishing autonomous from controlled motivation within the same model, the findings further indicate that the association between teacher emotional support and students’ psychological well-being should be understood in relation to both psychological need satisfaction and the form of students’ motivational regulation. Overall, the study provides a more differentiated Self-Determination Theory–based account of the relationship between teacher emotional support and student psychological well-being in higher education.

## Data Availability

The raw data supporting the conclusions of this article will be made available by the authors, without undue reservation.
